# Impact of Urbanization on Health and Well-Being in Ghana. Status of Research, Intervention Strategies and Future Directions: A Rapid Review

**DOI:** 10.3389/fpubh.2022.877920

**Published:** 2022-06-28

**Authors:** Doris Akosua Tay, Reginald T. A. Ocansey

**Affiliations:** Regional Institute for Population Studies, University of Ghana, Accra, Ghana

**Keywords:** urbanization, health and well-being, Ghana, urbanization health, public health, urbanization threats, health risks, urbanization policies

## Abstract

**Introduction:**

Ghana like other African countries is facing multiple health threats due to expansion of urban populations. Globally, the urbanization phenomenon has received considerable attention and modest steps have been undertaken to address it. Ghana is stalling on implementation of policies and interventions targeted at alleviating the menace.

**Objectives:**

This review examined research evidence, interventions, and policies relating to urbanization and threats to health and well-being of people living in Ghana. The review focused on three areas including urbanization threats to health and well-being, health risks associated with urbanization, and interventions and policies.

**Materials and Methods:**

The search spanned from year 2000 to February 2022 covering documents related to urbanization, health, and well-being. Databases used for the search include African Journals Online, Annual Reviews (Biomedical, Life & Physical sciences, Social Sciences), BioMedCentral, BioOne, BLDS digital library, Cambridge University Press, ClinicalKey, CINAHL, University of Ghana Digital Collections/UGSpace, JSTOR, Medline and Wiley Online Library.

**Results:**

Environmental risk factors, urban planning, water-related, behavior-related, and socioeconomic factors were important urbanization threats to health and well-being. Health risks identified include airborne diseases, waterborne diseases, malaria, and non-communicable diseases such as hypertension and lung cancer. Additionally, there is evidence of non-implementation and/or non-enforcement of existing interventions and policies.

**Conclusion and Recommendation:**

Evidence from this rapid review shows that urbanization impacts on health and well-being of people in Ghana. Urbanization threats that expose populations to health risks could be reduced through commitment to implementation, surveillance and monitoring of policies and interventions. Communities and individuals must be equipped to take control of their health and well-being.

## Introduction

Urbanization is causing a change in health of populations through the social environment, physical environment, delivery and access to healthcare (1). Countries are unable to cope with the rapid pace of urbanization in providing essential services (1). Urbanization also, may mutate the epidemiology of infectious diseases risking rapid spread (2). This can be observed in the recent Covid-19 pandemic. Urbanization therefore exacerbates disease transmission and disrupts provision of quality health services (3). Health improvement is dependent on infrastructures, technologies and guidelines that deliver clean sanitation and water (4). However, despite efforts made, cities are plagued with severe water shortages (4). Thus, increasing vulnerability of populations to health risks especially for those living in poor communities.

Urbanization is associated with risks of both communicable and non-communicable diseases (3, 5). People dwelling in urban areas have increased risk of mental disorders through stressors linked with congestion, increased crime, decline in social support and environmental pollution (6). Health risks related to urbanization have been categorized according to diseases that affect slums and urban poor, sedentary lifestyle related diseases, and diseases that travel beyond socioeconomic borders and affect all groups of urban dwellers (7).

African countries have not been able to implement effective population health interventions to meet the growing challenge of rapid urbanization (5, 8). Urbanization in Africa varies across countries and it is impractical to provide one solution that aims at solving problems for all (8). Solutions therefore must be contextualized to be successful.

An indispensable medium through which urbanization affects health and well-being is through air pollution. Outdoor and indoor air pollution together causes 8 million deaths a year internationally, it is the chief cause of childhood pneumonia and the foremost risk for childhood asthma (9). In Ghana air pollution is responsible for 28210 deaths (9). For example in the Greater Accra region alone, air pollution caused 1,800 deaths in 2017 (9). Exposure to pollutants such as heavy metals, ammonia emissions, black carbon, sand and mineral dust are closely related to morbidity and mortality (9).

Poor sanitation, a product of urbanization, is a major risk factor for morbidity and mortality. Poor sanitation is a menace in cities in Ghana and it undermines the livelihoods of dwellers (10). Policies developed to tackle sanitation issues such as the National Plastics Management policy are yet to see results (11). Rainfall and inadequate pipe-borne water supply in urban Ghana also affects sanitation due to inefficient water supply and drainage systems. Thus, dwellers are exposed to increased burden of water-borne diseases such as cholera and kidney diseases (12–15).

How urbanization associated health risk factors are tackled in Ghana is unclear. Thus, a conscious exploration of evidence pertaining research, policy documents and interventions would be key to understanding the urbanization phenomenon and then forge ways to address them. Therefore, this paper attempted to explore evidence pertaining to urbanization, health, and well-being in Ghana. The evidence was quarried focusing on three areas as follows, urbanization threats that affect health and well-being, health risks associated with urbanization, and interventions and policies.

## Materials and Methods

### Literature Search

Rapid review was chosen to produce a timely synthesis while following systematic review methods as suggested by Grant & Booth (16). Databases used for the search include African Journals Online, Annual Reviews (Biomedical, Life & Physical sciences, Social Sciences), BioMedCentral, BioOne, BLDS digital library, Cambridge University Press, ClinicalKey, CINAHL, University of Ghana Digital Collections/UGSpace, JSTOR, Medline and Wiley Online Library. These databases were used because they were more likely to have the relevant studies that would address the objectives. Mendeley was used to manage citations. Documents were exported to Mendeley. It identified duplicates which were deleted.

### Search Strategy

The search strategy entailed two phases. Specific search terms were used during the first phase. Search terms include urbanization and health Ghana; urbanization urban health well-being Ghana; urbanization threats that impede health and well-being Ghana; urbanization; and urbanization Ghana. These terms generated literature from the databases. The second phase included specific policy documents, websites, organizations, and citations search. These include urbanization, environment, and health related policies; organizations and websites such as World Health Organization, and Climate & Clean Air Coalition.

### Additional Search Criteria and Screens

Additional search filters included ‘Ghana', and this yielded more research conducted in Ghana. Screening was done to identify documents and research from a range of literature dating from 2000 to February 2022.

### Eligibility Criteria

**Figure 1:**
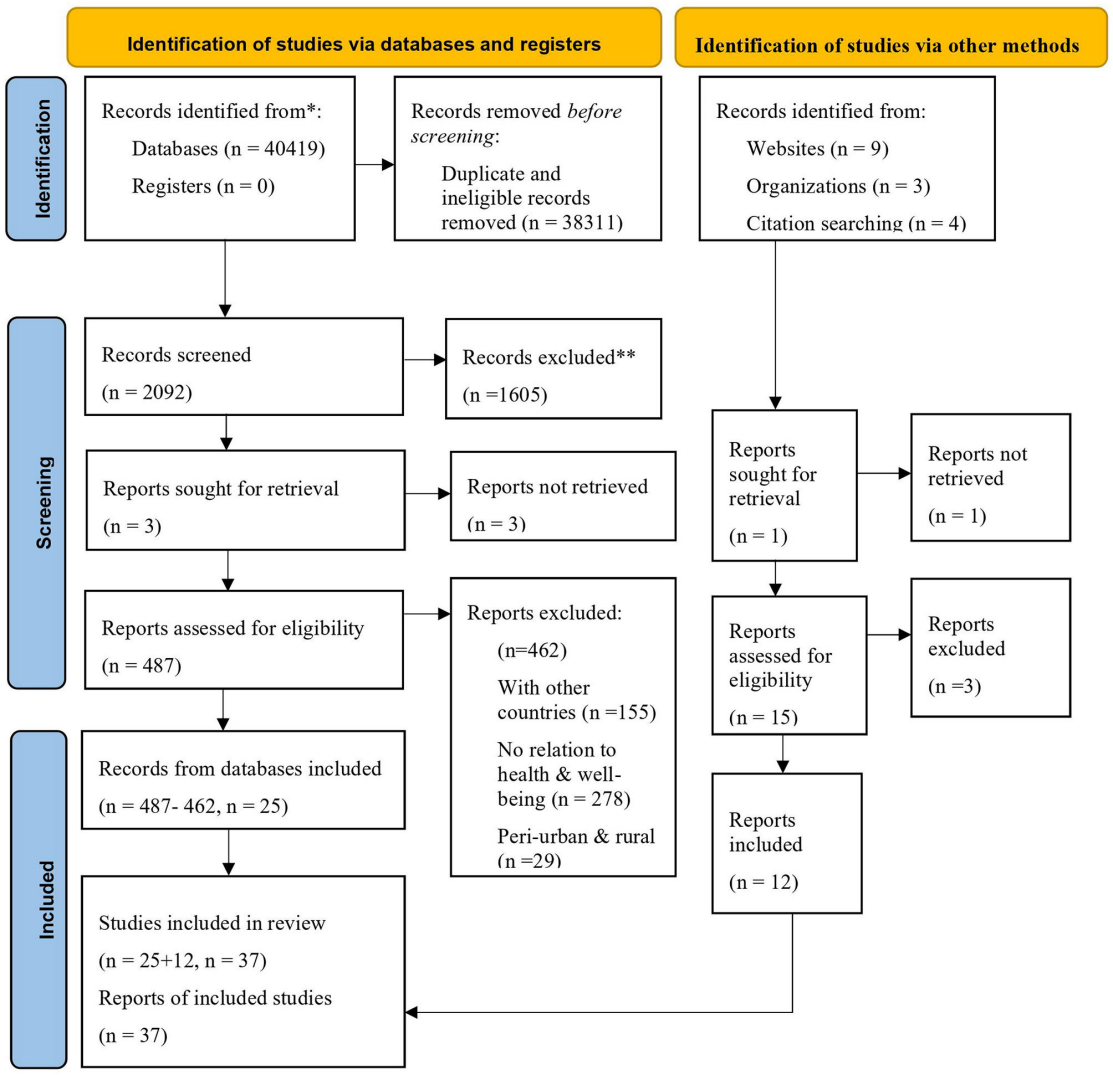
PRISMA flow chart of screening.

To be included in the review studies must be conducted in an urban area in Ghana and in English. Articles, research, and reports must include either urbanization with health and well-being, urbanization with health or urbanization with well-being. Published data and gray literature were included. The review excluded studies conducted in both urban and rural communities, peri-urban, rural, and sub-urban areas. Studies conducted on urbanization only with no linkage to health or well-being were also excluded. The inclusion and exclusion of documents involved three steps. The first step was to screen titles that comprised the search terms. The second step was to read the abstract for relevance and the last step was to read the entire document if it had an element of the review objectives. PRISMA 2020 flow diagram was used to report search details (17) as displayed in Figure 1. Total number of items identified through databases were 40,419. Screened records were 2,092 after 38,311 items made up of duplicate and ineligible records were removed. Studies from databases that met the inclusion criteria for the review were 25. Identification of documents through other methods were 16 of which 3 were excluded, one report was not retrieved, and 12 met the inclusion criteria. Nine records were from websites, 3 from organizations and 4 citation searches. In all, 37 studies both from databases and other search methods met the inclusion criteria and were reported on. See Appendix 1 for included studies.

### Screening Protocol and Biases

Two experts engaged in the review process. A study protocol to guide the process of the review was developed (see Appendix 2). Bias in this review is the exclusion of articles that were not published in English and older articles before year 2000 which may have important results relevant to the objectives. Although this review was interested in Ghana, inclusion of materials that were in other countries could have allowed for comparability. These, however, were not possible because of the time constraints to complete the review.

## Results and Discussion

The evidence obtained from the rapid review revealed three dominant foci including urbanization threats to health and well-being, health risks associated with urbanization and interventions/policies (Table 1). Table 1 provides a summary of the dominant foci number of documents retrieved and their associated risk factors or threats.

**Table 1:** Classification of focus areas by number of documents and associated risk factors/threats.

**Focused Areas**	*Number of documents retrieved	**Risk factors/Threats**
Focus #1: Urbanization threats to health and well-being	20 (42.6%)	1.Environmental risk factors 2.Water-related factors 3.Urban planning factors 4.Socioeconomic factors
Focus #2: Health risks associated with urbanization	13 (27.7%)	1.Malnutrition 2.Malaria 3.Diarrhea 4.Type 2 diabetes 5.Airborne diseases 6.Waterborne diseases 7.Hypertension 8.Lung cancer 9.Stroke 10.Chronic obstructive pulmonary disease 11.Acute lower respiratory infections
Focus #3: Interventions and policies	14 (29.8%)	1. There is evidence of non-implementation and/or non-enforcement of existing interventions and policies

^*^Although 37 documents were obtained from the rapid review (Appendix 1), some documents (n=10) were classifiable under more than one focus area. Thus, the total number of documents as shown in this Table equals 47.

### Focus #1: Urbanization Threats That Affect Health and Well-Being

Twenty documents (42.6%) dealt with urbanization threats that affect the health and well-being of residents. Areas covered include environmental risk factors, behavior-related, water-related, urban planning, and socioeconomic threats. Evidence from the documents retrieved revealed structural inadequacies relating to poor waste disposal and sanitation practices that are detrimental to health.

Air pollution is the country's number one environmental risk to public health and it is the 6th out of 19 risks of death (18). Household and ambient air pollution creates exposure to heart attack, lung cancer, stroke and childhood pneumonia among children (19). Insecticide resistance and the distribution of *Culex* species, a pathogen vector that assists in the transmission of malaria (20) is augmented by urbanization. This consequently surges the incidence and prevalence of malaria.

The review also identified issues related to urban sprawl. Urban sprawl is a growing phenomenon, and it produces a lack of basic household amenities, potable water supply, crime, anomic social behaviors, sanitation, and health issues (21–24).

Approximately 1 in 10 urban households lack a toilet facility (21). Most people (73%) depend on facilities that are shared by households or fee-paying and accessible to all (25). For sewered facilities Accra, Tema, and Kumasi are the only cities with a sewage network and of these, 23,000 connections are in Tema with only 1,100 in Accra (25). Practice of open defecation is a menace. Forty-five percent of the population in Accra practice open defecation (26). This may have resulted in an extensive spread and elevated levels of fecal pollution in public and private spaces and food supply (27). Exposure to fecal contamination occurs through open drains, markets, flood zones, and urban agricultural areas (27). Among children transmission is through eating uncooked food (27).

Cities in Ghana have issues with solid waste management (22, 24, 28, 29) and this is related to sociocultural beliefs, low sense of responsibility toward solid waste management, and weak enforcement of regulations. Landfills and dumpsites are major risk factors to health and well-being of people dwelling in urban areas (30, 31). Fecal sludge management services by municipalities and private operators offer emptying services (25). However, about 72 percent of the sludge produced in Accra and 43 percent in Kumasi are untreated before disposal into the sea and environment (25).

There is high level of mercury in water bodies in urban areas. This predisposes communities to food insecurity. Fish population is reduced resulting in a surge in nutritional deficiencies, and economic difficulties. The high levels of mercury also affect mental and physical health and well-being (32). A study observed that although Sakumo II (a lagoon) supports fish and crab populations, it is a repository for domestic and industrial waste (33). This poses an important threat to the health and quality of life of people through consumption of its products. The wetlands accrue pollutants such as heavy metals which makes it hazardous for regular ingestion (33) and an important cause of NCDs.

Other water-related factors include access to potable water and flooding. A disconnect exists between water service delivery systems and urbanization due to unplanned extension of settlements (32). High population densities in urban areas are linked to flooding. Rapid extension of sealed-off surfaces for instance, increases the incidence of flooding in Accra (28). This is because of increased impervious surfaces that decreases the absorption of storm water runoff (28). Expansion of built environment in relation to roads, parking lots and other impervious surfaces prevent water infiltration (28). Flooding is a major water-related threat that exposes people to water-borne and infectious diseases (28), mortality, and loss of livelihoods. People with a previous experience of diarrhea linked to flooding in urban areas, are observed to have a higher perceived risk of recurrence (34). This is likely to affect their mental health.

Socioeconomic factors associated with urbanization affects the health and well-being of residents. Low household income, high unemployment, fewer years of education, undesirable life events, large household size, and number of children are variables that produce high psychological distress and high crime rate within cities in Ghana (35, 36). Property crime rate in the urban cities is on the rise due to poverty and combinations of the observed characteristics (36). Furthermore, the review suggest that women in Ghana's cities are economically challenged and their roles have been confined to household heads who engage in menial economic activities to fend for their families (37).

Low standard housing facilities, lack of sanitation facilities (22, 23) and other amenities are linked to high prevalence of child malnutrition, malaria, and diarrhea (23). The level of deterioration of hygiene facilities is related to malaria deaths (31) while poor infrastructure and sanitation remain the highest risk factors to health and well-being (31).

### Focus #2: Health Risks Associated With Urbanization

Documents that addressed this area were 13 (27.7%). Health risks associated with urbanization include both infectious and non-infectious diseases. The review evidence suggest that urbanization in Ghana risks people's lives to pandemics and malaria because of poor urban planning system (38, 39). Non-communicable diseases such as lung cancer, stroke, ischemic heart disease, chronic obstructive pulmonary disease, and acute lower respiratory infections are serious health risks associated with high household air pollution (40).

In urban Ghana food security and food availability have influenced the dietary patterns of dwellers. Two prominent dietary patterns were identified, and these include, ‘purchase dietary pattern' and ‘traditional dietary pattern.' A purchase dietary pattern which involves the ingestion of high amounts of sweets, rice, protein-rich foods, fruits, vegetables and low ingestion of plantain are risk factors for type 2 diabetes (41). Increased odds of type 2 diabetes is linked to people with a traditional dietary pattern who ingest high amounts of plantain, green leafy vegetables, beans, garden eggs, fruits and palm oil (41). These results show that unhealthy dietary pattern exposes urban residents to health risk such as type 2 diabetes. Modification of food preferences therefore would reduce acquiring type 2 diabetes. Sex, level of education of household head, household wealth quintile, and source of food also have linkages with dietary diversity (42).

Unhealthy and healthy dietary pattern were linked to children's age and household socioeconomic status (43). Four important dietary patterns were identified among school children in Accra (43). These include energy dense diet, starchy roots and vegetable-based diet, cereal grain and poultry, and fish and sea food (43). Overweight and obesity was mainly linked to energy dense diet (43). Inequality in food quality and consumption is related to socioeconomic status of children (44). Poorer children are vulnerable to food insecurity, unhealthy and cheap food, and narrow dietary diversity (44). Wealthier children, however, ingest processed and packaged foods rich in sugar but low in nutrients (44). These predisposes children to obesity, nutritional deficiencies, and probable stunted growth and development. Women in urban areas have high rates of obesity and overweight and this exposes them to health risks such as hypertension, type 2 diabetes, cardiovascular diseases and other NCDs (37). Women dwelling in urban communities have a high prevalence of presumptive hypertension linked to high age, obesity, and a lower age than 50 years at menopause (45). Increasing age, obesity, parity of three or more children, no formal education and early age of menopause are some risk factors for hypertension among women (45) dwelling in urban communities.

Levels of malaria prevalence, transmission, and persistence vary in urban areas, and this is related to levels of urbanization in communities. For example, in Accra and Kumasi, mosquitoes were present in more polluted areas (20, 46) predisposing them to malaria. Resistance of *Anopheles* to insecticides, human infection-prone behavior, healthcare efficiency and *Plasmodium* resistance augments levels of malaria transmission and persistence (39). These factors if addressed through educational campaigns, promoting use of insecticide-treated bed nets, and larvicide applications disrupt the transmission pathway of Malaria (39).

Public health risks (27) such as intestinal infections, dysentery, cholera, hepatitis, typhoid, and skin diseases are associated to exposure to fecal contamination. Health risks associated with fecal contamination does not only affect households but the entire urban area through air-borne transmission (21).

### Focus #3: Interventions and Policies

The evidence from 14 documents (29.8%) showed there are interventions and policies aimed at addressing risk factors related to urbanization. However, the impact of such interventions and policies remains uncertain. For example, pollution appears in multiple policies and is managed by different institutions. It is unclear how regulation processes and monitoring of implementation are done.

The Urban Health and Short-lived Climate Pollutant Reduction Project (SLCP) is an intervention by the WHO (19). It aimed at encouraging strategies that results in a decline of air pollution through mobilization and empowerment of the health sector, and demonstration of the full range of health co-benefits that can be realized from the strategies at the city level (19). The Urban Health Initiative (UHI) also focuses on solid waste management to improve health by greening the waste sector (29, 30). The UHI is yet to see its success especially in the face of open waste burning and unsanitary dumps which have not been addressed.

Ghana's National Plan (NAP) focuses on mitigating SLCPs through multisectoral approach (47). As reported by (47), there is evidence of policy failures because of non-implementation of such policies (47). Additional health policies may not be the way forward but implementation and monitoring of existing policies (47).

Local Government Act 936 (2016) conserves and sustains the waste management districts in existence and includes the disposal of waste on land, such as discharge of effluent into a body of either still or running water (29). The Act also recentralizes the waste management department at metropolitan assembly level instead of the municipal and district assembly levels (29).

The informal sector participation in solid waste management, which is a survival strategy for practitioners has been unused by authorities (48). It is suggested that private sector waste management such as ‘Kaya bolas,' itinerant and stationary waste collectors, resource merchants, and small-scale recycling industries be integrated in current solid waste management policies for sustainability (48). This is expected to reduce the phenomenon of unmanageable solid waste which threatens population health and well-being. Apart from environmental contextual factors that impede effective management of solid waste in urban communities, inadequate infrastructure and waste collection processes is responsible for ineffective management (24). Aside from solid waste management, issues also exist in the provision of potable water, toilet facilities, and sanitation. These factors risk urban residents to ill-health and low quality of life.

A study analyzed three interventions aimed at alleviating flooding and poor solid waste management in Accra (28). They include construction of retention ponds, storm drain widening and community led solid waste management (28). Construction of retention ponds in the middle and upstream of the Odaw river is expected to gain direct economic benefits of prevention and reduction in damages caused by flooding (28). Indirect economic benefit is expected through less transport and business interruption (28). Storm drain widening is expected to reduce likely flood damages by 12 percent, prevention of mortality and health risks from enteric diseases, avoid productivity losses and reduce diarrheal disease by 30 percent (28). Community based solid waste management will remove waste, produce a clean environment, improved health and decreased flooding (28).

The Community-Based Health Planning and Service (CHPS) which was originally started in rural communities has been implemented successfully by health workers in urban communities (49). This will mean an improvement in health and well-being among the urban population. However, impediments such as lack of first aid medicines, and the Integrated Management of Neonatal and Childhood Illness program that has not been instituted affect its sustainability (49).

Kumasi, compared to Accra has almost eradicated an urbanization threat, open defecation (26). Although approximately 40 percent of the people depend on public toilets, Kumasi has been successful through an integrated approach to urban waste management especially by private sector potential (26). In 2018 Kumasi passed a Bylaw that required all houses to have toilets and this includes penalties for landlords who violated it (50). Accra in January 2022 implemented ‘Operation Clean Your Frontage' which is yet to see results.

Ghana's Vision 2020 (1996–2000) policy aimed at turning Ghana into a middle income country by 2020 (51). Stipulated in this policy were strategies to address health in both urban and rural communities through tackling of waste and sanitation, waste management and pollution (51). Although Ghana achieved the goal of a low middle-income country, it stalled on sanitation, solid waste management and pollution issues (51).

The National Environmental Sanitation Policy (NESP) (2010) aims to develop and maintain a clean, safe, and pleasant physical or natural environment (52). Complementary activities include provision and maintenance of sanitary facilities, and others which are yet to see success (52). Most of the principal components such as collection and sanitary disposal of wastes, inspection and enforcement of sanitary regulations, control of rearing and straying animals, and cleansing of thoroughfares, etcetera have not been fully implemented (52). The policy has no directive to address outdoor and indoor air pollution, a crucial factor of urbanization and a major cause of morbidity and mortality.

The Health and Pollution Action Plan (2019) launched in 2018 targets five pollution risks factors which affect health (53). Indoor air pollution, outdoor air pollution, contamination, exposures to soil pollution from heavy metals and toxic chemicals and occupational exposure to pollution are main pillars which will be worked on (53).

Policies and interventions aimed at reducing household air pollution include, the National Petroleum Authority (NPA) (2005, Act 691), WHO Household Energy Assessment Rapid Tool (HEART), Ghana Sustainable Energy for All (SE4ALL) Action Plan, National Policy on Promotion of Liquefied Petroleum Gas, 2010 Energy Policy and 2018 draft Energy Policy (54).

### Future Directions

Based on the evidence from the review, research must be scaled upwards and broadened to cover a wider geographical area in the country. This will foster a better understanding of the impact of urbanization in the country. Robust monitoring and surveillance systems are necessary across more urban cities to enable policymakers to appreciate the detrimental effects of urbanization and to propound policies to address them. More data must be generated on solid waste management methods and environmental loads.

Curbing the detrimental effects of urbanization requires a carefully guided implementation plan to ensure effective implementation of existing policies. This paper suggests a robust planning and implementation approach which focuses on concepts such as good urban planning to deal with urban sprawl, better housing options with amenities, access to clean water, and provision of safe environments. Construction of storm drains will reduce flooding together with enforcement of laws that prevent erection of structures on water ways. A multisectoral approach is also needed and institutions managing the same policies must come together and agree on monitoring processes to ensure successful implementation of the policies.

Clearance of landfills and dumpsites, enforcement of bylaws and provision of toilet facilities are essential to manage fecal contamination threats. Community led and private sector inclusion in waste management is expected to ameliorate solid waste management. Reintroduction of town inspectors and levies will help curb sanitation related issues. This way, the country would be better served.

Traffic congestion and environmental degradation pose eminent challenges and threats. Achieving physical planning goals must be integrated with the urgent need to control pollution and waste, improve air quality, and seek a better balance with the ecosystem within and outside of the urban cities.

An energy-optimizing model which focuses on the use of sunlight and wind can do well to improve the living environment. A conscious and culturally beneficial approach to urban design is likely to provide a satisfactory outcome. As with Accra and Kumasi, to rebuild a part of the neighborhood higher as in Jamestown, La, Teshie, Nungua etc., to consolidate the mixed land use patterns in super rise buildings and generate land for community use.

The bottom line is that Government and societies must take due responsibility to provide needed resources to ensure proper and effective planning and implementation of policies and/or interventions.

## Author Contributions

All authors listed have made a substantial, direct, and intellectual contribution to the work and approved it for publication.

## Conflict of Interest

The authors declare that the research was conducted in the absence of any commercial or financial relationships that could be construed as a potential conflict of interest.

## Publisher's Note

All claims expressed in this article are solely those of the authors and do not necessarily represent those of their affiliated organizations, or those of the publisher, the editors and the reviewers. Any product that may be evaluated in this article, or claim that may be made by its manufacturer, is not guaranteed or endorsed by the publisher.
