# Supplementary material for: Impact of Urbanization on Health and Well-Being in Ghana. Status of Research, Intervention Strategies and Future Directions: A Rapid Review
Source: Front Public Health. 2022 Jun 28;10:877920. doi: 10.3389/fpubh.2022.877920 (PMC9273841; doi:10.3389/fpubh.2022.877920)
Supplement: Supplementary file 1 [file Table_1.pdf]

Appendix 1- List of available evidence obtained from the rapid review

First Author	Year	Content Subject	Design	Summary of Key Findings
1. World Bank Group	2020	Environmental analysis	Secondary data analysis/review	Cost of environmental degradation to Ghana highest with air pollution, water pollution, gold mines, unmanaged solid waste, and other contaminated sites, and agricultural land degradation, deforestation, and overfishing.
2. Cavalieri	2017	Urban Health Initiative- air pollutant and their impacts	Case study	Household and ambient air pollution is the highest environmental health threat. Seasonal dust storms influence average annual air pollution.
3. Kudom	2015	Resistance status of *Culex* species to organophosphate and carbamate	Experimental	Mosquitoes were found in more polluted water bodies in Accra and Kumasi. Resistance to carbamate was observed in *Culex quinquefasciatus* populations. Mosquitoes were susceptible to organophosphate.
4. Brenyah	2013	Prevalence of malaria and effects of knowledge, attitudes, socioeconomic status, and preventive practices by residents	Experimental	Malaria prevalence and factors influencing transmission varies within communities in the same urban area.
5. Mudu	2021	Air pollution, health, and climate change impacts of different solid waste management practices	Secondary data analysis	Serious shortage of solid waste management data for Accra on management methods and environmental loads. Solid waste management responsible for significant emissions of greenhouse gases and critical air pollutants such as black carbon. Cessation of open burning will reduce 120 premature deaths by 2030 and

					increased composting and recycling produce significant decrease in carbon dioxide emissions.
6.	Fobil	2011	Relationship between malaria and infectious diarrhoea mortality and spatially varied neighbourhood environmental quality conditions	Secondary data analysis	Strong evidence of differences in relative mortality of malaria across urban environments of varying neighbourhood environmental conditions. Mortality related to diarrhoea showed no difference. There was no association between diarrhoea and environmental variables.
7.	Ghana Statistical Service	2014	2010 housing and population census report on urbanization	Census	Pipe-borne water is not available to every urban household in Ghana. Only 1 in 4 urban households have access to water closet facilities at household level with 1 in 10 households not having any form of toilet facility. The latter use bushes and beaches. Limited use of solid waste disposal by collection. High dependence on charcoal and wood as household cooking fuel.
8.	Oteng-Ababio	2019	Urbanization of Wa municipality, its synergism in risk accumulation, uncertainties, and complexities.	Mixed method case study.	Solid waste management in Wa is skewed with people in high class residence engaging in proper practices while resident in low-income communities is underserved with solid waste infrastructure. Evidence of inefficient potable water supply system and chronic sanitation situation with high levels of diarrhoea.
9.	Badasu	2011	Relation between housing and associated facilities and amenities, and child nutritional and health outcomes	Mixed method study design	High prevalence rates of malnutrition, malaria and diarrhoea among children were related to low standard housing facilities and dependence on public/shared sources of sanitation facilities and other amenities. Those children had higher rates of stunting and underweight. Children who lived in houses that did

					not have water closets were 4 times likely to be wasted than children who had access to such amenities.
10.	Lissah	2021	Managing urban solid waste; Perspectives and experiences of municipal waste company managers and supervisors	Phenomenological study design	Three main factors that influenced waste management include organizational, community, and contextual factors. Organizational factors include organizational capacity, resources, and expertise. Community aspects include socio-cultural beliefs influencing negative attitude of residents and a low sense of responsibility towards solid waste management. Contextual factors include rules, regulations, and their enforcement.
11.	Oteng-Ababio	2020	Analysis of how construction of retention ponds, storm drain widening, and community-led solid waste management can overcome urbanization challenges	Secondary data analysis	Construction of retention ponds results in direct economic benefit of prevention and reduction in flood damage. Storm drain widening reduces possible flood damages by 12%, diarrheal disease, and productivity loss. Community-led solid waste management results in benefits through a cleaner environment, improved health, and reduced flooding.
12.	WHO	2021	Urban Health Initiative	Intervention and review	Ending waste burning, expanding compost, and recycling programmes, and capturing landfill gas reduces emissions of greenhouse gases short-lived climate pollutants compared with business-as-usual activities. This is expected to improve health, reduce air pollution, and reduce climate change.
13.	Tiberghien	2016	Sanitation planning processes in developing countries that are making good progress in provision of sanitation services to all.	Case study	Kumasi has almost eradicated open defecation unlike Accra and the entire country. Access to improved household sanitation is very low with 40% of the residents depending on public toilets. Kumasi improved sanitation and management of both solid and liquid waste through private sector potential and expansion of capacity to rising demand and increase levels of service.

14.	Robb	2017	Pathways of exposure to fecal contamination	Microbiological analysis	Evidence of widespread and high levels of faecal contamination in both public and private domains and food supply. The main faecal exposure pathway for children in the household is through ingestion of uncooked food.
15.	Mansour	2017	Situation analysis of the urban sector in Ghana	Secondary data analysis	Ghana failed to achieve MDG for sanitation by reaching only 15% instead of its target of 54% coverage. With a marginal increase of 7% in urban areas. Entire families live in single rooms rented compound houses that lack basic sanitation and water supply. Slum upgrading projects has been implemented in low-income areas. These include construction of major roads and drains, provision of public toilets at strategic locations, and World Bank-funded Greater Accra Metropolitan Area Sanitation and Water Project to support Ghana Water company to extend services to low-income areas and to develop onsite sanitation services.
16.	Mosello	2017	Urban water in Ghana	Case study	Ghana has improved access to water sources in urban centers, but progress has largely been through bottled/sachet water and public taps or standpipes, rather than piped water. Large compartments of urban communities where the poorest live continue to be unserved by the Ghana Water Company Limited. There is lack of integrated urban planning, political incentives prioritizing vote-winning investments, fragmented pro-poor interventions, and asymmetric information on inequalities and access to water services has reduced progress.
17.	Abu	2018	Experiences and future perceived risk of floods and diarrheal disease in urban poor communities	Household survey	Thirty percent of households had experienced a point of reference flooding (26th October 2011) and members reported of diarrhea within four weeks after the flood. Twenty percent of the households reported members experiencing diarrhea within four weeks after the flood although they did not experience the flood of 26th October 2011. Households in

					Agbogbloshie have higher perceived risk of diarrheal disease than those in James Town. This is attributed to sanitation interventions in Agbogbloshie which includes distribution of refuse bins and construction of alley pavements to allow free flow of rainwater.
18.	Gbogbo	2015	Analysis of concentrations of five heavy metals Cadmium (Cd), Arsenic (As), Mercury (Hg), Copper (Cu), and Lead (Pb) in the lagoon water, sediment, green algae, eight species of aquatic macrophytes, seven species of arthropods, and one species of fish.	Sample analysis	Concentration of Pb was lower than the detection limit, and Cu was detected only in the lagoon water and *Pheropsophus vertialis*. Cd ranged from 21±4 ppb in algae to 69±12 ppb in Typha domingensis and was higher than As and Hg. The highest concentration of As was 11.7±2.1 ppb in Pistia stratiotes whilst Hg was highest in lagoon water (4±2 ppb). Concentrations of Cd and Hg (in macrophytes) was higher than USA EPA guidelines This demonstrates that the wetland's resources were unsafe for regular consumption.
19.	Dzator	2013	Hard times and common mental health disorders in urban Ghana	Household survey	Hard economic times affects psychological well-being. Except for working in agriculture, maintaining employment reduces the feeling of hopelessness. Being employed also improves self-esteem and mental health. People working in agriculture exhibit strong feelings of distress.
20.	Owusu	2016	Extent to which crime and poverty can be correlated in urban Ghana	Household survey and qualitative	Low-class and high-class neighborhoods are relatively safe compared to middle-class neighborhoods. The safety of low-class neighborhoods is attributed to strong social cohesion and the existence of guardianship at all times of the day. Sexual and property offences are crimes related to poverty.
21.	Frempong-Ainguah	2018	Pathways in which individual socio-demographic factors, economic characteristics, and endowment influence	Cross-sectional study	Different levels of health status are evident among women based on age, educational level, wealth status, ethnicity, disease symptom experienced, and social networks kept. On average the women scored lowest on vitality or fatigue, bodily pain, and general health issues. Changing economic situations have seen women taking jobs outside homemaking and taking

			self-assessed health status among women		on roles as household heads and bread winners. These produces stress and affects their general health and well-being. There were high rates of obesity and overweight among the women.
22.	Anaafo	2021	Linkages between urban planning and public health revealed by the COVID-19 pandemic	Secondary data analysis/review	The current urban planning system in Ghana is inadequate and exposes people to current and future disease pandemics.
23.	Savi	2021	Emerging property of malaria transmission and persistence	Qualitative and participatory-based system analysis and mapping	Forty-five determinants interplayed through 56 linkages and 3 subsystems namely, i. urbanization-related transmission such as deficient city planning and planning enforcement, inadequate housing conditions, and limited waste and sewage infrastructure leads to proliferation of mosquito breeding sites; ii. infection-prone behavior; and iii. healthcare efficiency and *Plasmodium* resistance.
24.	EPA	2018	The Greater Accra Metropolitan Area air quality management plan 2018	Longitudinal study, focused studies, and secondary data analysis	The plan includes development of emissions and ambient standards, analysis of emission source contributions, evaluation of air quality monitoring data, estimation of current and projected future health burden of air quality, establishment of goals and objectives for the plan, and development of a detailed implementation plan. Air pollution is generated through point sources (industrial sites), mobile sources (vehicles), and area sources from naturally occurring harmattan wind-blown dust and sea salts and man-made such as cook stoves and open burning of wastes. Air quality monitoring network shows high levels of particulate matter across locations, and this is expected to increase by 2030. Air pollution attributable mortality is 2800 deaths in 2015, 3100 in 2020 and 4600 in 2030.
25.	Frank	2014	Dietary patterns in Ghana and risk of type 2 diabetes	Case-control studies	Two dietary patterns identified to be associated with type 2 diabetes are ‘purchase’ dietary pattern and ‘traditional’ dietary pattern. Purchase dietary pattern

					was inversely associated with type 2 diabetes while the traditional dietary pattern increased the odds of type 2 diabetes. Participants in the highest quintile of purchase dietary pattern were younger, leaner, and of higher socioeconomic status while those in the highest quintile of the traditional dietary pattern were older, heavier, and more deprived.
26.	Codjoe	2016	Urban household characteristics and dietary diversity, an analysis of food security	Household survey	Fifty-three percent of the households ate foods prepared from outside the home, while 46.6% ate food prepared at home. About 13.4% of households have no expenditure on food and depend on their harvests from fishing and farming. Single member households, those with heads who have professional, technical, managerial, or clerical occupations, and those with heads that belong to the Mande ethnic group have the highest dietary diversity. Households in the poor and richest wealth quintile have higher dietary diversity than those in the poorest wealth quintile. Households with heads with high educational level, and those who eat food prepared outside the home have higher dietary diversity than those with lower education and those who eat home prepared food.
27.	Ogum	2018	Associations between dietary pattern and background characteristics among school age children (9-15 years) in urban Ghana	Cross-sectional study	Four dietary patterns that explained 53.2% of differences in the diet of children include energy dense, starchy root staple and vegetables, cereal-grain staple and poultry, and fish and seafoods. Child obesity/overweight is associated with energy dense food pattern while starchy root with vegetable dietary pattern is negatively associated with overweight/obesity.
28.	Stevano	2020	Urban food security and consumption among school children	Survey, qualitative focus group	Dietary diversity is associated with wealth. Inequality in food quality and consumption among children is partially linked to seriocomic status. Food insecurity and narrow food diversity is a problem for the urban poor. Consumption of packaged and processed food

				cuts across wealth groups because they are desirable, affordable, and accessible.
29. Duda	2007	Assessment of blood pressure in urban women	Community-based survey	Out of 1328 women 309 have a positive review of systems for hypertension and only 52.3% were using anti-hypertensive drugs. Risk factors for elevated blood pressure include age 50 years and above, BMI of 30 or more, parity of 3 or more children, menopause before 50 years, high fasting blood glucose and fasting cholesterol, no formal education, and first-degree family history of hypertension. Income level, diet and activity had no relation to hypertension among the women. Young age, high parity, normal BMI, and fasting LDL-cholesterol lower than normal were protective factors.
30. Republic of Ghana	2018	National action plan to mitigate short-lived climate pollutants (SLCPs)	Intervention	Sixteen SLCP mitigation measure across seven sectors were identified and prioritized. The execution of the 16 measures is expected to decrease methane, black carbon, carbon dioxide emissions and lead to considerable swift health, agriculture, and climate benefits.
31. Oteng-Ababio	2012	Role of the informal sector in solid waste management	Qualitative design	The role of the informal sector is seen through waste collection, recovery, and recycling to merchandising. Their activities generate economic linkages for society, lower cost of recycling for the communities, and impact positively on the environment.
32. Nwameme	2018	Health care personnel reaction towards the implementing community-based health planning and services (CHPS) in impoverished urban communities	Qualitative methods	Despite challenges faced, health personnel have successfully implemented the urban CHPS concept. Impediments with implementation include absence of provision of first aid drugs and the execution of the Integrated Management of Neonatal and Childhood Illness (IMNCI).

33.	Aquaya	2019	Sanitation policies, practices, and preferences	Reviews, transect walks, qualitative methods.	Public toilets are dominant in low-income areas in Kumasi. Residents prefer private, low volume flush toilets that require little space and infrequent emptying. The city's durable pits and flat topography decreases the risk for flooding. A fecal sludge facility is available but not fully operational. Current and ongoing sanitation programs focus on improved sanitation facilities and wastewater treatment.
34.	Republic of Ghana	1995	Ghana vision 2020	Policy	Strategies to address health in both urban and rural communities through tackling of waste and sanitation, waste management and pollution.
35.	Ministry of local government and rural development	2010	Environmental sanitation policy	Policy	Complementary activities include provision and maintenance of sanitary facilities. Principal components include collection and sanitary disposal of wastes, inspection and enforcement of sanitary regulations, control of rearing and straying animals, and cleansing of thoroughfares.
36.	Republic of Ghana	2019	Health and pollution action plan	Policy	Targets five pollution risks factors which affect health, and these include indoor air pollution, outdoor air pollution, contamination, exposures to soil pollution from heavy metals and toxic chemicals and occupational exposure to pollution.
37.	Edwards	2021	Evidence-based strategies to reduce the burden of household air pollution	Secondary data analysis	Policies and interventions aimed at reducing household air pollution include, the National Petroleum Authority (NPA) (2005, Act 691), WHO Household Energy Assessment Rapid Tool (HEART), Ghana Sustainable Energy for All (SE4ALL) Action Plan, National Policy on Promotion of Liquefied Petroleum Gas, 2010 Energy Policy and 2018 draft Energy Policy.
