# Supplementary material for: Impact of Urbanization on Health and Well-Being in Ghana. Status of Research, Intervention Strategies and Future Directions: A Rapid Review
Source: Front Public Health. 2022 Jun 28;10:877920. doi: 10.3389/fpubh.2022.877920 (PMC9273841; doi:10.3389/fpubh.2022.877920)
Supplement: Supplementary file 2 [file Data_Sheet_1.pdf]

Appendix 2- Study Protocol

1. Scope, title, and objectives.
2. Type of Review- use systematic review. This was replaced by rapid review because the time frame fell short of systematic review requirement.
3. Background/Scope- Status of urbanization, urbanization and health and well-being at the global level and Africa, Ghana *(how urban population has grown over the years since first census);* and other relevant information. Linkages between urbanization and health.
4. Rationale and Objectives- To determine the impact of urbanization on health and well-being in Ghana through existing research and examine interventions that have been put in place and its effectiveness to address existing and emerging health risks.
5. Thematic Areas drafted- The review focuses on these thematic areas: *(SUGGESTED)*

- Urbanization threats that impede good health and well-being
- Health risks associated with urbanization
- Forms of interventions, and its impact
- Policies addressing urbanization and health *(this should be in the interventions)*
- Focus of research *(because of status of research in the topic)*
- Environmental factors associated with urbanization that affects health and well-being
- Social factors of urbanization that impacts on health and well-being.

Thematic areas were reviewed as presented in the report. i. Urbanization threats to health and well-being, ii. Health risks associated with urbanization, and iii. Interventions and policies.

6. Methods

Period- 2013 – 2022 *(2013 was when Ghana launched its first national urban policy).* To help us know the situation after the policy was launched. However, we were interested in the situation before the policy, so the year was modified to start from 2000 to capture literature pre, during, and after MDGs.

Eligibility- Studies that falls within review period. Urban communities excluding rural communities. Published and unpublished data *(unpublished data was later excluded)*. Studies carried out on Ghana. Articles, reports, research. Studies on urbanization and health and well-being. Must be in English. **Exclusion-** Studies outside Ghana or on Ghana with other countries, and before review period, studies on urbanization only, studies in rural communities and studies that cover both urban and rural areas.

Search Terms

Urbanization and health; urbanization; urbanization threats that impede good health and well-being Ghana; urbanization health well-being urban Ghana. Additional search, Ghana.

Data Base- Google, Google scholar, ScienceDirect, African Journals Online, Annual Reviews (Biomedical, Life & Physical sciences, Social Sciences), BioMedCentral, BioOne, BLDS digital library, Cambridge University Press, ClinicalKey, CINAHL, University of Ghana Digital Collections/UGSpace, JSTOR, Medline, Wiley Online Library.

Total-

Records identified from:

Databases (n =)

Registers (n =)

Records removed before screening: (n =)

Duplicate records removed (n =)

Records marked as ineligible by automation tools (n =)

Records removed for other reasons (n =)

Records screened (n =)

Records excluded (n =)

Reports assessed for eligibility-
